# Advancement and New Trends in Analysis of Pesticide Residues in Food: A Comprehensive Review

**DOI:** 10.3390/plants11091106

**Published:** 2022-04-19

**Authors:** Shadma Wahab, Khursheed Muzammil, Nazim Nasir, Mohammad Suhail Khan, Md Faruque Ahmad, Mohammad Khalid, Wasim Ahmad, Adam Dawria, Lingala Kalyan Viswanath Reddy, Abdulrahman Mohammed Busayli

**Affiliations:** 1 Department of Pharmacognosy, College of Pharmacy, King Khalid University, Abha 61421, Saudi Arabia; 2 Department of Public Health, College of Applied Medical Sciences, Khamis Mushait, King Khalid University, Abha 61412, Saudi Arabia; ktahir@kku.edu.sa (K.M.); mosukhan@kku.edu.sa (M.S.K.); 3 Department of Basic Medical Sciences, College of Applied Medical Sciences, Khamis Mushait, King Khalid University, Abha 61412, Saudi Arabia; nnasir@kku.edu.sa; 4 Department of Clinical Nutrition, College of Applied Medical Sciences, Jazan University, Jazan 45142, Saudi Arabia; mfahmad@jazanu.edu.sa (M.F.A.); abdulrahmanbusayli@gmail.com (A.M.B.); 5 Department of Pharmacognosy, College of Pharmacy, Prince Sattam Bin Abdulaziz University, P.O. Box 173, Al-Kharj 11942, Saudi Arabia; drkhalid8811@gmail.com; 6 Department of Pharmacy, Mohammed Al-Mana College for Medical Sciences, Safaa, Dammam 34222, Saudi Arabia; wasima@machs.edu.sa; 7 Department of Public Health, College of Health Sciences, Khamis Mushait Campus, King Khalid University, Abha 61412, Saudi Arabia; aabdelqader@kku.edu.sa; 8 Department of Public Health, College of Health Sciences, Saudi Electronic University, Abha 61412, Saudi Arabia; lv.reddy@seu.edu.sa

**Keywords:** food, pesticides, toxicity, maximum residue limits, analytical techniques, QuEChERS, chromatographic

## Abstract

Food safety is a rising challenge worldwide due to the expanding population and the need to produce food to feed the growing population. At the same time, pesticide residues found in high concentrations in fresh agriculture pose a significant threat to food safety. Presently, crop output is being increased by applying herbicides, fungicides, insecticides, pesticides, fertilizers, nematicides, and soil amendments. A combination of factors, including bioaccumulation, widespread usage, selective toxicity, and stability, make pesticides among the most toxic compounds polluting the environment. They are especially harmful in vegetables and fruits because people are exposed to them. Thus, it is critical to monitor pesticide levels in fruits and vegetables using all analytical techniques available. Any evaluation of the condition of pesticide contamination in fruits and vegetables necessitates knowledge of maximum residue levels (MRLs). We set out the problems in determining various types of pesticides in vegetables and fruits, including the complexity and the diversity of matrices in biological materials. This review examines the different analytical techniques to determine the target analytes that must be isolated before final consumption. Many processes involved determining pesticide residues in fruits and vegetables and their advantages and disadvantages have been discussed with recommendations. Furthermore, MRLs of target pesticide residues in fruit and vegetable samples are discussed in the context of data from the literature. The review also examines MRLs’ impact on the international trade of fruits and vegetables. Accurate, sensitive, and robust analytical procedures are critical to ensuring that pesticide levels in food products are effectively regulated. Despite advances in detection technology, effective sample preparation procedures for pesticide residue measurement in cereals and feedstuffs are still needed. In addition, these methods must be compatible with current analytical techniques. Multi-residue approaches that cover a wide range of pesticides are desired, even though pesticides’ diverse natures, classes, and physio-chemical characteristics make such methods challenging to assemble. This review will be valuable to food analysts and regulatory authorities to monitor the quality and safety of fresh food products.

## 1. Introduction

All nations’ primary priority is to increase food production, since the world’s population is predicted to reach over 10 billion by 2050. Evidence suggests that the global population is adding 97 million people each year. An alarming report from the Food and Agricultural Organization (FAO) of the United Nations indicates that the world’s food supply has to rise by 70 percent to meet the growing population’s demand [1]. Consequently, the ever-increasing global population has placed significant strain on the present agricultural system, which may meet food demands while using the same resources, such as land, water, and other natural resources, that are already available. Presently, crop output is being increased by applying herbicides, fungicides, insecticides, pesticides, fertilizers, nematicides, and soil amendments. These compounds entered the picture primarily with the advent of synthetic pesticides in 1940, when organochlorine (OCI) insecticides were first utilized for pest control [2].

The relevance of food quality has become a severe concern owing to the extensive usage of pesticides. Farmers may have a traditional understanding of agriculture, but they lack technical knowledge of pesticides, their applications, and safety considerations, leaving them susceptible [3]. Pesticide use has increased globally during the last decade due to the world’s growing population and fast urbanization. There is a possibility that these residues in food, whether active pesticide components, metabolites, or breakdown products, will harm the human body. Thus, it is vital to inform customers about the possible risks of pesticide use [4]. According to the literature, there is a danger associated with various pesticides with diverse modes of action. Long-term pesticide exposure causes neurological impairments and depression, diabetes, and respiratory disorders, such as rhinitis [5]. In addition to providing a measure of food quality, residue analysis may be used to identify and prevent potential health concerns and determine the quantity and persistence of chemical pollution within the environment. The European Union Commission (EU) [6], Codex Alimentarius Commission (CAC) [7], and Gulf Cooperation Council (GCC) are responsible for the MRLs in 50 countries and where 23 countries follow their unique set of MRLs, such as Food Safety and Standard Authority of India (FSSAI) [8].

Pesticides are classified into organic and inorganic pesticides by their chemical nature. Synthetic pesticides are categorized as cyclodiene, organochlorine (OCs), carbamate, organophosphate (OPs), synthetic pyrethroids, triazole, and nicotinoid, and they are extensively utilized owing to their benefits in the field [9]. Pesticide levels in any given location are highly dependent on pesticide application intensity and crop kinds. Given pesticides’ mixed effects, maximum selectivity should be our goal. Pesticides most likely contaminate fruits and vegetables, mainly citrus fruits, grapes, and potatoes [10].

Pesticide residues in fruits and vegetables must thus be examined urgently, since they may increase the risk of different illnesses to human health. Any evaluation of the condition of pesticide contamination in fruits and vegetables necessitates knowledge of MRLs. The current study focuses on worker-validated residue detection techniques, and each analytical parameter is addressed for method validation. We set out the problems in determining various types of pesticides in vegetables and fruits, including the complexity and the diversity of matrices in biological materials. This review examines the different analytical techniques to determine the target analytes that must be isolated before final consumption. Many processes involved in determining pesticide residues in fruits and vegetables and their advantages and disadvantages have been discussed with recommendations.

Furthermore, MRLs of target pesticide residues in fruit and vegetable samples are discussed in the context of data from the literature. The review also examines MRLs’ impact on the international trade of fruits and vegetables. Accurate, sensitive, and robust analytical procedures are critical to ensuring that pesticide levels in food products are effectively regulated. Despite advances in detection technology, effective sample preparation procedures for pesticide residue measurement in cereals and feedstuffs are still needed. In addition, these methods must be compatible with current analytical techniques. This review will be valuable to food analysts and regulatory authorities to monitor the quality and safety of fresh food products.

## 2. The Methodology of the Literature Review

The literature has been searched using PubChem, Google Scholar, ScienceDirect, Web of Science, and the Saudi digital library. The following keywords and phrases were used to explore the databases, such as pesticides residues, pesticides in fruits, pesticides in vegetables, safety considerations in food, pesticide exposure, residue analysis, potential health concerns, European Union Commission, organic and inorganic pesticides, pesticide residues in fruits and vegetables, pesticide contamination in fruits and vegetables, analytical parameter, diversity of matrices in biological materials, analytical techniques, MRLs of target pesticide residues in fruit and vegetable, current analytical techniques, regulatory authorities to monitor the quality and safety of fresh food products, leading existence of pesticides, maximum residue limits (MRLs) and toxicity, impact of MRLs on the trade of vegetables and fruits, analytical techniques, detection of pesticide residues, extraction methods, liquid–liquid extraction, solid-phase extraction, QuEChERS, liquid-phase micro-extraction, matrix solid-phase dispersion, chromatographic detection approaches, gas chromatography, liquid chromatography, liquid chromatography–tandem mass spectrometry, optical screening methods for pesticide residue in food matrices, ambient desorption/ionization mass spectrometry methods, and impacts of pesticide residues removal.

## 3. Vegetables and Fruits with Leading Existence of Pesticides

Environmental Working Group (EWG), a nonprofit organization, publishes an annual list of the 12 vegetables and fruits with the most pesticide residues. According to their pesticide residues, strawberry, spinach, kale, nectarine, apple, grape, peach, cherry, pear, tomato, celery, and potato are named dirty dozen. These commodities had the highest pesticide levels of the year [11]. The Pesticide Data program was released in recent years by the United States Department of Agriculture (USFDA). The maximum number of pesticides found in various fruits and vegetables is shown in Table 1.

Source: EWG’S 2021 DIRTY DOZEN LIST (https://www.ewg.org/foodnews/dirty-dozen.php (accessed on 7 February 2022)).

Key findings of EWG’s analysis are represented below:

- Only 2% of the samples tested positive for avocados and sweet corn pesticides, respectively.
- The first seven clean fifteen crops are sweetcorn, avocados, onions, pineapples, papaya, eggplant, and sweet peas, which tested positive for three or fewer pesticides on a single sample.

Pesticide residue concentrations in food and feed should be kept as low as feasible while providing the necessary protection to the treated crops or animals. However, conducting supervised experiments on them is challenging due to the wide range of crops. Consequently, the trials are focused on significant, representative commodities; the residue levels recorded in these commodities are used to predict residue levels in ‘minor crops’ within the same commodity group. There are many reasons why residue dispersion varies so much across trees and plots and between pesticide-treated areas. For example, there are significant differences in how much pesticide is used, applied, and spread out in the canopy and leaves [12,13,14,15,16,17,18,19,20,21].

In 2019, 96,302 samples were analyzed, with 96.1 per cent falling below legally permissible limits. However, for the subset of 12,579 samples analyzed as part of the EU-coordinated control programme (EUCP), 98% were within legal limits. Twelve food products were randomly collected and analyzed: apples, head cabbages, peaches, spinach, lettuce, strawberries, oat grain, tomatoes, wine (red and white), barley grain, and swine fat and milk of cow.

The results of the samples analyzed:

- A total of 6674 (or 53 percent) of the samples were residue-free.
- A total of 5664 or 45% contained one or more residues in concentrations below or equal to permitted levels.
- A total of 241 (or 2% of the total) included residues above the legal limit, with 1% resulting in legal action.

Exceedances rose for strawberries (1.8% to 3.3%), head cabbages (1.1% to 1.9%), wine grapes (0.4% to 0.9%), and swine fat (0.1% to 0.3%). As of 2016, no exceedances were found in cow’s milk.

## 4. Maximum Residue Limits (MRLs) and Toxicity

Above all, safe food should have an appropriate nutritious value and contain the least possible amounts of substances that could be hazardous to health. It is, therefore, crucial to monitor pesticide residues in fruit and vegetables [22]. It is becoming more vital for food and trade policy in the early 21st century to consider how pesticide MRLs interfere with agricultural commerce. The MRL refers to the number of pesticide residues in a particular food after its manufacture following Good Agricultural Practices (GAP). Pesticides are administered to crops, and residues are gathered to calculate maximum residue level (MRL) [23]. It is illegal to sell, import, or export products that have residual levels over the MRL [24]. Pesticide residue levels vary widely across nations, potentially affecting commerce. The issue of pesticide residue levels varies across countries and is developing and interfering with agricultural trade, which is problematic considering the extensive use of pesticides in agricultural output internationally [25,26]. Pest infestations have reduced agricultural output, leading to insecticides to control pests. Farmers throughout the globe have relied on pesticides to keep weeds, pests, and illnesses from decimating their harvests for decades [25,27]. Pesticide residues from crops are typically detected in foods, causing persistent health effects in people who eat them [28]. Agricultural usage of nitrogen fertilizers and pesticides has increased in the last 30 years. There are around 600 active substances of pesticides now in use, but appropriate toxicologic data for 100 of them are available. Humans are often exposed to pesticides, causing acute and chronic health impacts. It is the cause of acute and chronic neurotoxicity (fumigants, fungicides, insecticides), chemical burns (anhydrous ammonia), lung damage, and infant methemoglobinemia. Pesticide exposure has been related to several malignancies, including hematological tumors. In addition, pesticides have been linked to immunologic abnormalities in reproductive and developmental consequences. The toxicological effects of pesticides are not limited to a few chemical classes [26].

In Beirut, Lebanon, researchers examined the risk of pesticide residues in 49 plant-based foods. This study used the quick, easy, cheap, effective, rugged, and safe QuEChERS method to extract pesticides and evaluate them using liquid and gas chromatography–tandem mass spectrometry. The results have shown that 58 (32.2%) of the 387 specimens had residues. Over 50% of positive tests for 14 residues exceeded FD EU maximum residue limits. According to the conclusion of this study, pesticide residues in food may be safely consumed in the vast majority of cases [29]. As a result, increased efforts are required to minimize or eliminate human exposures whenever feasible. The food safety authorities establish maximum residue limits (MRLs) to control crop pesticide residues. MRLs are set to achieve the most incredible residue level predicted under regular agricultural practice by collecting the data for a specific pesticide. Unfortunately, Ordinary people do not know the exact meaning of MRLs and oversee that food containing pesticide residues above MRL level could not be consumed. According to the general population, pesticide residues in food represent more significant health hazards than other dietary dangers. International parties, such as the European Union (EU), Codex Alimentarius Commission (Codex), and North American Free Trade Agreement (NAFTA), have attempted to harmonize pesticide legislation by providing MRLs. Still, globally, these limits remain variable [30]. In addition, various organizations dealing with safety have different views on the safety of domestic processing. In contrast, the *Codex Alimentarius* recommends cleaning raw materials as often as necessary to eliminate dirt or other contaminants before use [31]. Most pesticide maximum residue levels established by the Codex give a high degree of protection. However, the MRLs are set well below harmful levels for humans; thus, food residues are more significant than the MRL [32].

Most nations have unique legislative requirements for MRLs, such as the Food and Drug Administration (FDA) in the US, Pest Management Regulatory Agency (PMRA) in Canada, and the European Commission (EC) in Europe [32]. In addition, the WHO and FAO created and endorsed MRLs. Pesticide residues on fruits and vegetables may theoretically be decreased by processing, and their amount is anticipated by physio-chemical factors, including solubility, hydrolytic rate constants, residue placement, volatility, and octanol–water partition coefficients. However, there is a paucity of specific data in practice, notably on dietary component interactions. Pesticide registration in several countries includes testing for pesticide residues in food stored and processed. Fresh fruit and vegetable post-harvest pesticide residue persistence and distribution have recently been the subject of detailed investigations. The research examined whether pesticide MRLs can preserve public health in 114 countries. Human body weight and food intake were used to analyze the average food intake rate and the theoretical maximum dose intake (TMDI) for each nation to convert worldwide MRLs to TMDIs. This research identified and analyzed 14 common pesticides and 12 standard agricultural products. A healthiness threat study revealed that over 30% of the calculated TMDI values exceeded the accepted daily intake (ADI). Although typical pesticide MRLs in foods were lacking, additional human exposure routes, such as water, soil, and air, were not evaluated. It was determined that globally standardized pesticide MRLs might significantly reduce pesticide exposure and limit human health concerns [33].

A study was conducted to analyze the pesticide residues in cowpea and maize. Thirty-seven pesticides comprising nine pyrethroids, fifteen organochlorines, and thirteen organophosphorus pesticides were reviewed in cowpea and maize samples. The study results showed that MRLs p,p-DDD, p,p-DDE, β-endosulfan, and β-HCH were increased in cowpea and maize samples. It indicates a high risk of chronic toxicity for these food eaters [28]. MRL is not toxicological. The research was conducted to assess the toxicological properties of diuron and chlorpyrifos residues connected with toxicological aspects in rabbits. MRLs are legal limitations placed on certain active ingredients and dietary combinations. The MRL is a trading standard developed by international and national organizations to regulate residues in global food commerce. MRLs may be used to ensure that the pesticides are only being used following GAP. MRLs are based on good agricultural practices and the lowest consumer exposure necessary to protect vulnerable consumers. Several nations have good operating procedures for optimum pesticide application, including farmer and operator training. Reasonable assurance of no damage is the standard used by the FDA to determine limits for pesticide/food combinations under the 1996 Act. In this case, the probability of developing cancer for a lifetime is less than one in a million. Pesticides prohibited in Canada or the US may be legal in Europe [34]. The MRLs for fruits and vegetables in the United States, Canada, and Europe are shown in Table 2. For example, organochlorines lindane was prohibited in Canada in December 2004. Also required is that residue limits be expressly confirmed to be safe for children before implementation. These limits are generally based on the maximum residues found following good agricultural practice, provided that these levels are toxicologically acceptable.

## 5. Impact of MRLs on the Trade of Vegetables and Fruits

MRLs were unheard of three decades ago, but they have gained popularity recently. Pesticide MRLs are perhaps the first thing producers should consider when managing pests. Agricultural goods, for example, may be sold to around 200 nations from the perspective of US exporters; nevertheless, it seems that there are 200 different sets of legislation governing these countries’ MRL policies [36]. A rising number of US producers and exporters, who ship abroad one in every three planted acres, are concerned about the absence of internationally agreed-upon criteria for pesticide residues. This risk-based methodology of evaluating the threat of chemical and likely exposure through food intake is the legal norm in the United States and many other nations, including Canada, Japan, New Zealand, and Australia, following WTO requirements [37]. Much research has focused on MRLs and commerce [38,39,40,41,42,43,44,45]. MRLs and agricultural commerce are becoming essential in the early 21st century. Countries with different pesticide residues could have a significant impact on trade. Stricter MRLs with US partners have a variable influence on US–EU commerce. The estimations show that a stricter MRL regulation reduces US exports of vegetables and fruits to European Union countries by 13.8 percent. The study’s findings show a large discrepancy in MRL rules across numerous important US overseas markets for fruits and vegetables, notably between the EU and Trans-Pacific trade partners [46].

The European Union (EU) has one of the strictest pesticide policies. As one component of the regulatory framework, Regulation (EC) No 396/2005 sets harmonized maximum residue levels for pesticide residues in food and feed in the European Union to ensure high levels of consumer protection. Under certain circumstances, a notification in the Rapid Alert System for Food and Feed (RASFF) is released for pesticide residues exceeding a specific or a default maximum limit [47]. EU pesticide policies are clear regarding the trade of vegetables and fruits inside and outside the European Union (Regulation (EC) No. 1107/2009; Regulation (EC) No. 396/2005) [30,48,49,50]. Most agriculture policymakers and economists believe that new 21st-century trade barriers, such as Sanitary and Phytosanitary (SPS) requirements, are unclear. There is a chance they could be more trade-distorting than tariffs, which are usually used to protect against imports [51,52,53,54,55]. SPS regulations are supposed to make it easier for people to make and sell things by ensuring plants, animals, and people stay healthy and giving people a way to tell if something is good or bad [53]. On the other hand, these policies can obstruct commerce, whether purposefully or accidentally. The 2016 National Trade Estimate on Foreign Trade Barriers Report clarifies that trade obstacles between the United States and other countries may be exacerbated by nontransparent and discriminatory SPS policies [56]. The WTO’s SPS Agreement allows countries to establish their criteria, but they must be science-based, nondiscriminatory across nations with comparable circumstances, and non-protectionist [57,58].

In contrast, data suggest that nations utilize SPS policies to safeguard local producers [59]. In global and bilateral trade discussions, there is no agreement on the effect of SPS policies on trade and no uniform framework for addressing SPS policy changes [59]. Our research addresses this gap in the literature. Some regulations facilitate trade while representing important product quality and safety enhancements [60,61]. So far, data on SPS trade consequences have been equivocal [62,63]. According to WTO statistics, more than 60% of nontariff trade barriers (NTBs) notices are connected to SPS, while 36% are related to MRLs [64,65]. The problem of different levels of pesticide residues in different countries is growing, causing issues for agricultural trade. It could have a significant impact because pesticides are used worldwide in farming [52]. An increased empirical study investigates the link between tighter tolerance limits and increased trade flows [39,40,42,43,44,45,60,66,67].

SPS and MRL rules are intended to encourage trade, yet they may stifle commerce purposefully or unwittingly. Unintentional differences in MRLs may have significant commercial repercussions. Foreign and local food producers will face increased compliance costs, higher consumer prices, and even the possibility of halted commerce if a product is denied at a port of entry due to an overly tight tolerance established by the importing nations [68]. So, it lowers food exports and commercial possibilities. Most industrialized nations built their MRL systems, recognizing that MRLs reflect food safety requirements and rising consumer concerns for the environment and human health [52]. Studies demonstrate that tighter MRLs for plant items in importing nations impede trade. Others are developing national MRLs. Having national MRLs creates regulatory heterogeneity and may operate as a trade barrier. Various studies concluded that harmonizing laws would improve trade for specific import requirements.

## 6. Analytical Pesticide Testing or Detection of Pesticide Residues

Pesticide usage in food and vegetables has risen worldwide in recent decades, resulting in significant worry about the long-term effects on human health. As a result, pesticide residues in food and vegetables must be tightly controlled and monitored to ensure consumers’ health [69,70]. Accurate, sensitive, and robust analytical procedures are critical to ensuring that pesticide levels in food products are effectively regulated. Different strategies for extracting and detecting pesticides in various fruits and vegetables have been established, ranging from conventional extraction to sophisticated detection. Pesticide residues in vegetables and fruits are examined in two steps: first, extracting and cleaning the target analytes from the matrix, and, second, determining the target analytes [71]. Conventional extraction and detection approaches, on the other hand, are described in detail in some previously published studies. Still, no widely approved standard procedures exist for extracting pesticides in labs [72].

### 6.1. Pretreatment and Extraction Methods

Extraction separates pesticide residues from the rest of the sample by utilizing a solvent. The extraction technique follows a standardized approach that involves the liberation of the required analyte from the matrices. The cleaning process pertains to a phase or group of stages in the analytical technique. Most of the possible interference co-extracts are eliminated using various physical and chemical means. Moisture is removed during pesticide analysis, and the remaining co-extractives are separated using a variety of separation processes. What can be extracted from the substrate depends on the extraction method and solvent type. The extraction technique should be designed to remove pesticides from the matrix (high efficiency) quantitatively, not induce chemical changes in the pesticide, and utilize affordable and cleaned instruments. Several approaches, such as solid-phase microextraction (SPME), QuEChERS (rapid, easy, cheap, effective, robust, and safe) extraction, solid-phase extraction (SPE), accelerated solvent extraction, microwave-assisted solvent extraction, supercritical fluid extraction, and liquid–liquid extraction (LLE), may be utilized for the extraction of target analytes from the material [9].

Analytical quality requirements, such as precision, sensitivity, and selectivity, have been met to suit the need for any particular analysis. Pesticide residue analysis may be performed on various substrates, including liquids such as water, fruit juices, and bodily fluids (partitioning), and solids such as soil, meat, and green plant materials (use of sorbent). The extraction solvent selection is used based upon the substrate and pesticide type. According to the studies, acetonitrile, ethyl acetate, dichloromethane, methanol, and toluene are the primary widely utilized solvents for evaluating pesticides in fruits and vegetables. However, the solvent must have good solubility for the pesticides and low solubility for co-extractives, and it should not chemically alter or react with the pesticide. In certain circumstances, solvent mixes are utilized to enhance the recovery of the procedures. [73,74].

The extraction technique begins by preparing sub-samples. The initial material comprises 0.5-to-2-kg samples that are cleaned and then homogenized in a mixer. Further extraction is performed on homogenized sub-samples weighing between 0.5 and 100 g. Due to its simplicity, liquid–liquid extraction (LLE) and solid-phase extraction (SPE) were first employed in the extraction process. However, owing to the microscale extraction procedure used by QuEChERS, its application has expanded significantly during the last decade [75]. Extracting organic compounds from various matrices (such as food and biological samples) is a lengthy procedure that requires much time. Still, the QuEChERS approach saves analysis time, minimizes analysis stages, and provides excellent recovery by using fewer reagents. In the Quechers method, pH value is set at about 5. Mustapha F. A. Jallow et al. determined the pesticide residue levels in popular fruits and vegetables eaten in Kuwait. QuEChERS multi-residue extraction was used to evaluate 150 samples of various fresh vegetables and fruits for the presence of 34 pesticides, followed by GC/MS or liquid chromatography–tandem mass spectrometry (LC/MS). According to this manuscript [76], pesticide residues above the maximum residue limits (MRL) were detected in 21% of the samples, and 79% of the samples had no residues of the pesticides surveyed or contained residues below the MRL.

A comparison of extraction techniques was conducted in a couple of the reported methods. Afify, Attallah, and El-Gammal compared a method to the QuEChERS, ethyl acetate, and Luke extraction methods. However, the QuEChERS approach exhibited excellent recovery for none, medium, and polar across a 60–70% recovery range for all three types of polarities. The Luke approach had a substantial impact when it came to recovering non- and medium-polar chemicals. Furthermore, compared to the other procedures, the Luke approach was determined to need the least amount of cleaning. However, ethyl acetate caused considerable recovery only for the polar compounds [75,77,78,79]. Overall, as per the literature, a lot has changed in the extraction process over the last few years. It has made the sample preparation procedure easier, cut the time it takes to analyze the sample, and uses fewer toxic solvents.

#### 6.1.1. Liquid–Liquid Extraction (LLE)

**Figure 1:**
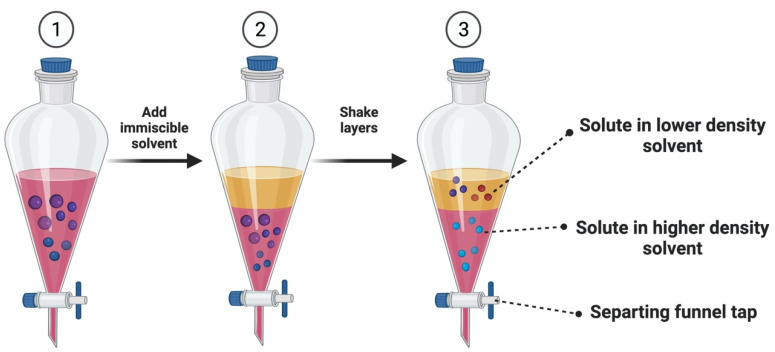
The processes in liquid–liquid extractions.

Liquid–liquid extraction (LLE), sometimes referred to as partitioning, is a separation technique that involves transferring a solute from one solvent to another that is immiscible or partly miscible with the first [80]. It is determined by the equilibrium distribution coefficient and depends on the analyte’s solubility in two immiscible solvents. The processes in liquid–liquid extractions are shown in Figure 1. In LLE methods, organic solvents, such as acetonitrile, chloroform, hexane, and 1,2-dichloromethane, are used to find pesticide residues in food and the environment because of their ability to dissolve in a variety of immiscible solvents. The partition coefficient of the donor and acceptor phases determines its extraction effectiveness. In liquid–liquid extractions, medium-polarity solvents, such as ethyl acetate, reduce the polarity of a polar solvent while increasing the polarity of a nonpolar solvent. High-performance liquid chromatography paired with an ultraviolet-visible detector was used to optimize and confirm liquid–liquid extraction with low-temperature partitioning to measure aldicarb, carbofuran, and carbaryl in chocolate milk beverages and grape juice accordingly [81]. A study reported the examination of atrazine, ametryn, terbutryn, carbaryl, and chlorothalonil in beer, wine, and Ethiopian honey wine using LLE accompanied by high-performance liquid chromatography with an ultraviolet-visible detector. The study’s findings indicate that the proposed liquid–liquid extraction process is a highly selective and efficient sample preparation approach prior to quantitative measurement of the target analytes using HPLC with an ultraviolet-visible detector [82,83]. Another study reported that the technique of LLE with ethyl acetate was devised to determine mebendazole and its hydrolyzed and reduced metabolites in pig, chicken, and horse muscles. The results were examined using liquid chromatography–tandem mass spectrometry [84,85].

LLE is one of the most well-known and well-established procedures for pesticide extraction. It uses a variety of extraction solvents, including carbendazim (CB), thiabendazole (TB), and 6-benzyl aminopurine (6-BA), which is one of the most often utilized medium-polarity solvents for pesticide extraction from matrices [86]. LLE is dependable, versatile, and compatible with a wide range of equipment. For example, De Pinho et al. (2010) utilized acetonitrile and ethyl acetate (6.5 mL:1.5 mL) to separate chlorpyrifos, cyhalothrin, cypermethrin, and deltamethrin from honey samples. Acetonitrile was also employed as an extraction solvent for carbamates (aldicarb, carbofuran, and carbaryl) in water samples [87,88]. However, the use of organic solvents in LLE results in a considerable number of hazardous leftovers, the development of difficult-to-break-up emulsions, and the difficulty of automating the whole process, making liquid–liquid extraction a laborious, time-consuming, and expensive procedure.

#### 6.1.2. Solid-Phase Extraction (SPE)

Solid-phase extraction (SPE) is among the most extensively utilized packing column or cartridge extraction procedures because of its simplicity, speed, and capacity to handle many samples with excellent recovery [89]. In SPE, extracts are transported via the cartridge and adsorbed on solid-phase substances prepared and activated using water or organic solvent prior to use. The SPE is conducted before the selective retention of target analytes on an adsorbent packed in a disposable extraction of a mini-column. Based on their interactions, analytes are first adsorbed onto appropriate substances. After that, interferences are removed using a selective organic solvent, and the target analytes are eluted with a different solvent [90]. The solvent in SPE is determined by the pesticide’s molecular properties (ionic and nonionic). Methanol, acetonitrile, petroleum ether, dichloromethane, acetone, ethyl acetate, hexane, acetic acid, toluene, and cyclohexane are some solvents that have been employed in SPE [91].

Various SPE cartridges are employed for pretreatment and pesticide residue analysis in fruits and vegetables. A few techniques for determining pesticide residues have been published using the florisil column, C18 columns, and Envi-carb cartridges. C18 was employed in SPE material to avoid peak widening in the online SPE high-performance liquid chromatography system. An amino propyl (NH2) solid-phase extraction cartridge is utilized to remove lipid components at low temperatures. Planar molecules are retained and removed by GCB. Sugars, organic acids, and fatty acids were removed using the silica-bond TMA chloride (SAX)-PSA cartridge. A solid-phase extraction adsorbent based on multi-walled carbon nanotubes was initially developed to extract organophosphorus pesticides from fruit juices [92]. The GCB-PSA dual-layer SPE method removed fatty acid matrix components from various food matrices. Even though Envi-Carb or NH2-LC could not absorb both the pigment component and the polar materials (e.g., protein, sugar, etc.) of the berry matrix individually, their linked column could provide the most significant clean-up effectiveness and recovery both for nonpolar and polar pesticides [93,94,95]. The need to obtain meaningful results as the basis for determining the content of trace amounts of analytes has become the driving force behind the development of modern analytical techniques, including sample preparation techniques, such as SPE. Recently, great interest was aroused in using magnetic nanoparticles (MNPs) in SPE. These materials exhibit high selectivity and, in small amounts, can provide high recovery of analytes, even from large-volume samples. MNPs allow easy, rapid isolation of analytes using an external magnetic field. In magnetic SPE, these materials provide effective isolation and enrichment of the analytes from samples with complex matrices (e.g., biological, environmental, and food samples) [96,97,98,99].

Gel permeation chromatography–solid phase extraction–gas chromatography–tandem mass spectrometry has been proposed to simultaneously determine organochlorine pesticides in milk and milk powder samples. All organochlorine insecticides have a quantitative limit of 0.8 g per kilogram. They found that the average recovery rates ranged from 70.1 to 114.7 percent at three spiked concentration levels (0.8, 0.20, and 10.0 mg/kg), with residual standard deviations of less than 12.9 percent at all three levels. The proposed approach was successfully used to assess organochlorine pesticides in commercial dairy products [100]. The author discovered that, when the concentration of MeCN in the aqueous solution increased, the recoveries of OCPs placed on the SPE cartridge reduced. The recoveries varied from 95% to 103% when the MeCN content was equal to or less than 20% [101]. In a study, the determination of bispyribac sodium residues in rice was accomplished using solid-phase extraction in conjunction with high-performance liquid chromatography with a diode array detector [102]. Because the pH of analytes determines their stability, the pH of extracts is critical for ensuring good pesticide retention on the adsorbent. As a result, maintaining pesticide stability and increasing analyte absorption in the solid phase necessitates the use of an adequate pH. Researchers used1.0 M NaOH to bring the pH of orange, pineapple, apple, and grape juices to 6.0 to assure organophosphorus pesticides’ stability [103].

**Figure 2:**
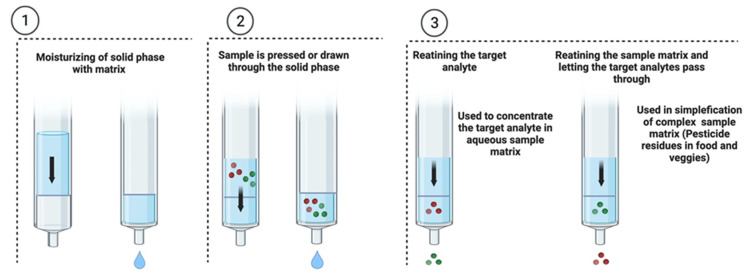
Solid-phase extraction procedures for the pesticide residue analysis in fruits and vegetables.

Researchers employed a multiwalled carbon nanotube (MWCNTs) as an SPE sorbent in a study to remove 36 pesticide residues from spinach and cauliflower. The LODs were discovered in the range of 0.1 to 5 g/kg, with recovery rates ranging from 57 to 108 percent when the relative standard deviation (RSD) was less than 12 percent. According to the findings, MWCNTs may have superior performance with high-polar pesticides [103]. The advantages of SPE procedures include more convenience, more superficiality, use of less solvent, shorter concentration times, gives more remarkable yield recovery, easier to automate, and does not result in emulsion formation. Furthermore, it can complete the whole sample preparation process without further treatments and provide the clean-up technique for various extraction methods [104]. In addition, a dispersive solid-phase extraction (dSPE) is relatively easier to operate than LLE, and it efficiently avoids the development of emulsions that often occur during LLE. The solid-phase extraction procedures technique for the pesticide residue analysis in fruits and vegetables is shown in Figure 2.

Additionally, SPE is a more practical and cost-effective process than GPC. SPE has become a standard approach for removing or concentrating pesticides in food samples; however, several elements still require refinement. For example, it is challenging to choose fast acceptable adsorbents and elution solvents to study multi-pesticide residues with a wide variety of physicochemical properties. In addition, the commercial SPE cartridges cannot be reused, which will significantly raise the experimentation cost.

#### 6.1.3. QuEChERS (Quick, Easy, Cheap, Effective, Rugged, and Safe Method)

Anastassiades et al., in 2003, first proposed the quick, easy, cheap, effective, rugged, and safe (QuEChERS) technique, which is a simple, rapid, and economical procedure for sample preparation in pesticide removal in food and vegetables. Many researchers employed this strategy due to its multiple benefits [105]. QuEChERS is a two-step procedure that begins with liquid–liquid extraction and ends with a diffusive solid-phase extraction clean-up. It usually depends on micro-scale extraction using MeCN, water absorption, and liquid–liquid partitioning employing MgSO4 and NaCl. The primary–secondary amine adsorbent is used in the QUCHERES clean-up stage of d-SPE. Chromatographic analysis eliminates the need for blending, filtering, significant volumes of solvent transfers, evaporation/condensation, and the required solvents transfers. In addition, the materials that have been pretreated with QuEChERS are clean enough to be examined with gas or liquid chromatography [106].

In contrast to the conventional QuEChERS method, the dry ice-partitioning QuEChERS technique was developed to determine pesticides in paprika by skillfully utilizing dry ice to endorse the separation of the upper MeCN layer without the salting-out effect and to evade probable deterioration of the thermal impact caused by the addition of MgSO4 and NaCl [107]. According to the latest research, the QuEChERS method produced favorable outcomes with a significant recovery (satisfactory ranges) of 72 pesticides in soy, carrot, silage, corn, tobacco, melon, cassava, lettuce, wheat, and rice, as well as 3 insecticides and 11 fungicides in strawberry by-products [108,109]. Furthermore, the upgraded QuEChERS approach, which uses a change in solvent properties, such as methanol and ethyl acetate, is currently employed in GC and LC detection, since it is highly appropriate for these instruments [110,111].

For pesticide multi-residue determination in green tea using LC-MS, a modified QuEChERS approach was designed and tested. The technique worked well in the concentration level of 0.01 to 1 mg kg^−1^. All pesticides could be measured at or below 0.01 mg kg^−1^. Lead acetate was initially used in conjunction with PSA and GCB to remove tannin, caffeine, and other tea colors, reducing matrix effects [26]. Another study on optimizing the clean-up stage of the QuEChERS technology in coffee leaf extracts evaluated 52 pesticides using LC-MS/MS. For this, the clean-up step of the QuEChERS technique was adjusted with several adsorbent combinations, yielding significant recovery (>70%) [112]. Pesticide levels in fruits and veggies eaten in Kuwait were determined employing QuEChERS multiple-residue separation guided by gas chromatography–mass spectrophotometer. Several pesticides were detected in 40% of the specimens, and four samples included more than four pesticide residues. Pesticide residues over the MRL were found in 21% of the 150 samples, whereas residues below the MRL were found in 79%. Aldrin was found in one apple sample that was below the MRL. Deltamethrin, imidacloprid, malathion, acetamiprid, diazinon, monocrotophos, and cypermethrin all exceed their maximum residue levels (MRLs) [76].

The QuEChERS approach, invented by researchers, does not need any cleaning. With a detection and quantification limit of 5 ng g^−1^ and 10 ng g^−1^, respectively, the suggested technique effectively identified 128 substances. In addition, the approach was straightforward to use and provided high recovery (70–120%), with an RSD of less than 20%. According to the researchers, the matrix impact was likewise found to be within the analyses’ limitations [113]. Recent research used a modified QuEChERS method that included the addition of acetonitrile and 0.1 percent formic acid, followed by a UHPLC-MS/MS analysis of 310 pesticide residue samples from brown rice, oranges, and spinach, and found that 87–89 percent of pesticides spiked at 10 ng g^−1^ met the criteria for acceptable concentrations set forth by the DG-SANTE guidelines [114].

A recent study examined the QuEChERS technology to identify pesticides in globe artichoke leaves and fruits materials. For the analysis of 98 pesticides in globe artichoke, a comparative investigation was conducted using QuEChERS, MSPD, and dispersive ethyl acetate. The results show that adding CaCl2 to the clean-up stage of the QuEChERS approach improved the dehydration of the samples and the formation of insoluble calcium salts with catecholic hydroxyls. GC-MS and LC-MS/MS were also used to detect the technique. In terms of LODs, the GC-MS and LC-MS/MS values ranged from 0.005 to 0.025 mg/kg and 0.003 to 0.015 mg/kg, respectively, with 70–120 percent recovery for both methods [115]. Sampling preparation using QuEChERS is a faster and less time-consuming process with minimal use of organic solvent. It has a promising future in residual pesticide analysis in foodstuffs because it minimizes the extraction and clean-up steps throughout sample processing and gives consistent quantitative findings.

#### 6.1.4. Liquid Phase Micro-Extraction (LPME)

Liquid-phase micro-extraction (LPME) is categorized into three parts in the sample preparation process: hollow-fiber liquid-phase micro-extraction (HF-LPME), dispersive liquid–liquid micro-extraction (DLLME), and single-drop micro-extraction (SDME). It is a simplified liquid-phase extraction process. The analytes are transferred from an aqueous phase to water-immiscible solvents in LPME. SDME was used in conjunction with gas chromatography–mass spectrometry to determine organochlorine pesticides in vegetable samples. It was used to determine the presence of OCPs in vegetable samples, and the recoveries ranged from 63.3 to 100%, with RSD ranging from 8.74 to 18.9%. In addition, exposure duration, agitation, organic drop volume, and organic solvent were regulated and adjusted parameters [116].

A novel approach depending on phase hollow-fiber liquid-phase microextraction has been devised to assess organophosphorus pesticides and some of their metabolites in two marketed products, one wheat flour and the second cereal-based infant meals, prior to gas chromatography–nitrogen phosphorus analysis. Ultrasound aided extraction with ACN containing 1.25 percent (*v*/*v*) formic acid was used to extract the samples first. Then, following evaporation and reconstitution in Milli-Q water, the HF-LPME approach was used, using 1-octanol as the extraction solvent and a desorption step in ACN, which significantly increased the technique’s efficacy [117].

A method for detecting pyrethroid pesticides in apple juice, vegetable juice, orange juice, kiwi juice, and peach juice has been developed. It uses two-phase hollow-fiber liquid-phase microextraction and gas chromatography–mass spectrometry to obtain the pesticides residue. A rotatable-centered cube central composite design was used to investigate the characteristics that impact extraction effectiveness. The response surface graphs indicated that agitation at 480 rpm, an extraction period of 41 min, and a NaCl concentration of 3% (*w*/*v*) resulted in the best separation. The optimization findings revealed that agitation speed, extraction duration, and ionic strength were significant factors in the extraction procedure. Limits of detection were found to be between 0.02 and 0.07 ng/mL, while limits of quantification were found to be between 0.08 and 0.10 ng/mL [118].

#### 6.1.5. Matrix Solid-Phase Dispersion (MSPD)

MSPD combines extraction and cleaning into a single phase, resulting in a technique that is simple, quick, and low in sample waste and solvent use. It is a standard sample preparation technique for detecting pesticides in food samples, such as vegetables, oil, fruit, biota, fish, and eggs. MSPD comprises sample homogenization, cellular disruption, exhaustive extraction, fractionation, and adsorbent clean-up. In the MSPD, solvents such as MeCN, methanol, EtAc, DCM, and combinations are utilized. Elution solvent composition and volume are critical for pesticide desorption from the adsorbent and interference absorption on the SPE column. For example, hexane was ineffective for eluting pyrethroid and organochlorine insecticides from alumina columns; however, EtAc proved efficient [119].

MSPD is quick, affordable, and may be performed under moderate experimental parameters (room temperature and atmospheric pressure), providing adequate efficiency and selectivity and, as a result, reducing environmental pollution and improving the safety of workers. The researcher conducted a comparison analysis for 105 pesticides using the modified QuEChERS and MSPD methods. According to the study’s outcomes, the improved QuEChERS approach outperformed MSPD in terms of extraction efficiency. SPME extraction was carried out via liquid partitioning with acetonitrile saturated with petroleum ether, followed by MSPD utilizing aminopropyl as the sorbent substance and a florisil cartridge for final clean-up. QuEChERS approach comprised liquid–liquid partitioning with acetonitrile, followed by dispersive solid-phase extraction and additional clean-up using GCB, PSA, and C18 sorbent, with final analysis utilizing fast liquid chromatography–electrospray time-of-flight mass spectrometry (LC-TOF/MS). The LODs were determined at concentrations ranging from 0.2 to 10 g/kg, with a 70 to 130 percent recovery rate. Although the MSPD extracts are clean enough for direct experimental analysis, fatty matrices generally need a second cleaning step. In addition, the MSPD approach is challenging to automate and may be time-consuming when working with larger samples [120].

#### 6.1.6. Other Extraction Methods

While QuEChERS extraction procedures are often used to extract and clean pesticide residues in food items, other approaches are also used in labs as substitutes for pesticide residue extraction. For example, GPC, also known as size exclusion chromatography, is a potent clean-up process initially employed in the 1970s to extract and clean-up up pesticides. GPC uses a molecular size-based separation method and elutes giant molecules first, followed by smaller ones. GPC is widely suggested to clean up extracts produced from biological samples, since it is the best approach for multi-residue pesticide analysis. However, there are several drawbacks, such as that the GPC needs specialized equipment, which is prohibitively expensive. For example, researchers used GPC to determine the concentration of 100 pesticides from samples of 240 fruits and vegetables. The eluent for the GPC method used was ethyl acetate-cyclohexane [121].

According to the findings, using a magnetic core dramatically increases the extraction of target analytes with high efficiency while using eco-friendly solvents. A study reported a novel extraction approach utilizing liquid–solid extraction combined with magnetic solid-phase extraction based on Pst/MNPs to detect pyrethroid residue, which is unique compared to existing methods [122]. Molecularly imprinted polymer (MIP)-based sensors circumvent the present limitations of classic detection methodologies. They hold significant promise for effective, low-cost, and narrow detection limit sensing employing smart nanoscale equipment. However, several disadvantages may arise due to MIPs’ lack of electrocatalytic activity and conductivity, limiting their use in the sensing sector. The incorporation of NPs and MIPs into innovative sensor chips has offered new avenues for quick rapid screening of pesticides. It was reported that, with this inclusion, the surface area of the nanocomposite had significantly been increased to promote the removal of the template from the polymer matrix and offered improved accessibility to recognition sites, rapid binding kinetics, and high binding capacity [123].

### 6.2. Chromatographic Detection Approaches

Several traditional analysis techniques are utilized for the second approach in pesticide analytical estimation, such as detecting or analyzing target analytes (pesticides) in foods. These techniques are high-performance liquid chromatography (HPLC), gas chromatography (GC), or more exclusive approaches such as gas chromatography associated with mass spectrometry (GC-MS), ultra-high-performance liquid chromatography–tandem mass spectrometry (UHPLC-MS/MS), and liquid chromatography associated with mass spectrometry (LC-MS). It is challenging to establish a pesticide detection technique in actual samples because of matrix interference. However, due to their sensitivity, separation, and identification capacities, GC and LC have become the most popular methods for detecting and quantifying pesticides in fruits and vegetables. Aside from this, various techniques for determining pesticide residue in actual samples have been utilized, such as capillary electrophoresis (CE) and enzyme-linked immunosorbent assay (ELISA) [124].

#### 6.2.1. Gas Chromatography (GC)

GC is a superior separation technology used in various investigations for volatile, readily vaporized pesticides. It is often used in conjunction with detectors, such as a flame ionization detector (FID) for the measurement of organophosphorus pesticides in onion, grape, and apple juices [125] or pyrethroid pesticides in vegetable oils [126]. According to most reported investigations, pesticide detection is accomplished using GC in conjunction with various detectors. Electron capturing detectors (ECD) [127], flame photometric detectors (FPD) [128], nitrogen phosphorus detectors (NPD), and mass selective detectors (MSD) [127] are utilized due to their sensitivity.

The flame photometric detector (FPD) was used in a study to identify 11 organophosphorus pesticide residues on mustard kale and cabbage samples [129]. The researchers also utilized the electron capture detector (ECD) to determine fenitrothion, chlorpyrifos-methyl, vinclozolin, and procymidone on peach [130]. For pesticide analysis, mass spectrometer detectors (MS and tandem MS) are equally preferred alternatives, shown in a study that includes monitoring of 381 specific pesticides in grapes by GC/MS-MS [131]. Another study reported the utilization of GC-MS to assess 35 regularly utilized pesticides [132]. A fused silica column with an internal diameter of 0.2 mm and a particle size of 0.25 μm is used for 95% of the GC chromatographic separations, with helium or nitrogen serving as the carrier gas. To examine pesticides, the authors documented the use of rapid gas chromatography combined with negative chemical ionization mass spectrometry [133]. Over the last couple of years, there has been an upsurge in the usage of polar pesticides (low persistence and high toxicity), making GC techniques less valuable. It is because polar pesticides are very volatile and have reduced thermal stability.

#### 6.2.2. Liquid Chromatography (LC)

In the examination of pesticide residues, a variety of liquid-chromatography-based approaches have been proposed, the majority of which are combined with ultraviolet (UV), photodiode array (PDA), diode array detector (DAD), and mass (MS) detectors. Gradient mode has been applied with the most often used stationary phase, octadecyl (C18), to reduce duration in multi-residue analysis. The majority of methods published in the literature for determining many pesticides in food utilize the HPLC approach in a reversed-phase system, utilizing gradient elution and a linear rise in the proportion of organic solvent. Isocratic elution is only employed on rare occasions [134]. On the other hand, HPLC analytical procedures are preferred as an efficient separation approach for high-polarity and nonvolatile extracted analytes. It may be used in conjunction with detectors, such as UV for pyrethroid residue analysis in fruits and vegetables or MS and tandem MS for determining triadimefon imidacloprid, diazinon, and malathion in fruit juices (pineapple, apple, orange, raspberry, and cherry) [135,136].

Currently, time-of-flight mass spectrometry (TOF-MS) is used with ultra-high-performance liquid chromatography (UHPLC) to identify 60 pesticides in 286 vegetable and fruit samples. In another study, UHPLC began using considerably smaller stationary-phase particle sizes (≤2 µm) than those employed in traditional LC (3–5 µm), in conjunction with tandem mass spectrometry (UHPLC-MS/MS), to identify 21 pesticides in sweet pepper and tomato samples [137]. However, more significant pressures must be used to obtain a smaller particle diameter. As a result, high-pressure pumps designed explicitly for UHPLC systems must provide a significant eluent flow rate in the column. Therefore, the UHPLC system is now the best option for chromatographic separation of multicomponent mixtures due to the chromatographic system’s substantially higher separation efficacy than standard HPLC [138].

#### 6.2.3. Liquid Chromatography–Tandem Mass Spectrometry (LC-MS/MS)

Pesticide residue detection using LC-MS is one of the most advanced analysis disciplines. It has been tested and implemented in this area in almost every form of ionization source and mass analyzer. It is usual practice to build LC/MS systems that conduct both multi-class and multi-residue analysis concurrently. The vast majority of these techniques have been specifically tailored to fruits and vegetables. There are few LC-ESI-MS/MS applications for pesticide analysis in baby meals. On the other hand, LC-QqQ-MS/MS has made it easier to determine more than 50 pesticides simultaneously, which is a lot [139].

Currently, gas chromatography–mass spectrometry (GC-MS, GC-MS/MS) and liquid chromatography–tandem mass spectrometry (LC-MS/MS) with electrospray ionization (ESI) are the two most common methods for multi-residue pesticide analysis in food today. Because they have high sensitivity and selectivity, they can be used to evaluate numerous pesticides from different chemical classes in extremely complex food matrixes in a single run. It is possible to differentiate thermolabile, nonvolatile, and underivatized compounds by LC/MS analysis. LC-MS can analyze a substantially more significant number of compounds than GC-MS. Tandem mass spectrometry (MS/MS) is a high-efficiency separation technology that consists of two coupled analyzers of the same or different kinds [139]. Collision-induced dissociation (CID) occurs when ions collide with neutral gas molecules is the most often utilized fragmentation method in liquid chromatography–tandem mass spectrometry systems. Integration of several kinds of analyzers is possible in various ways in LC-MS/MS. The most common types of analyzers used in tandem mass spectrometry for identifying pesticide residues in food are quadrupole–time-of-flight systems (Q-TOF), quadrupole–linear ion trap systems (Q-Trap), and triple quadrupole systems (QQQ) [140].

#### 6.2.4. Optical Screening Methods for Pesticide Residue in Food Matrices

However, chromatographic methods and mass spectrometry are costly, time-consuming, and need highly experienced staff, necessitating the search for simple, low-cost, quick, and on-site substitutes. Biochemical tests that employ antibodies or enzymes as identification components have typically been performed on microplates; ELISA is an excellent example of this kind of bioassay. ELISAs have been designed to detect pesticides in various food matrices, including OPs, neonicotinoids, and fungicides [141,142]. The significant breakthroughs in nanomaterials have boosted fluorescence detection even more. The identification of four OP pesticides, notably paraoxon, dichlorvos, malathion, and triazophos, was successfully shown by employing CdTe quantum dots as the fluorescent probe linked to an AChE-choline oxidase enzyme combination [143]. Colorimetry is the most basic and user-friendly optical detection technology; however, fluorescence (FL), surface plasmon resonance (SPR), and surface-enhanced Raman scattering (SERS) may often deliver more sensitive findings because of their selectivity and conjunction with nanomaterials. On-site pesticide residue detection may be made more accessible using portable handheld SERS instruments, which do not need sample preparation. On-site screening can also be carried out by combining optical screening assays with smartphones to be used everywhere. In light of the increasing globalization of the food industry, smartphone-based pesticides detection may be highly valuable for border inspections and field testing [144].

#### 6.2.5. Ambient Desorption/Ionization Mass Spectrometry Methods

**Figure 3:**
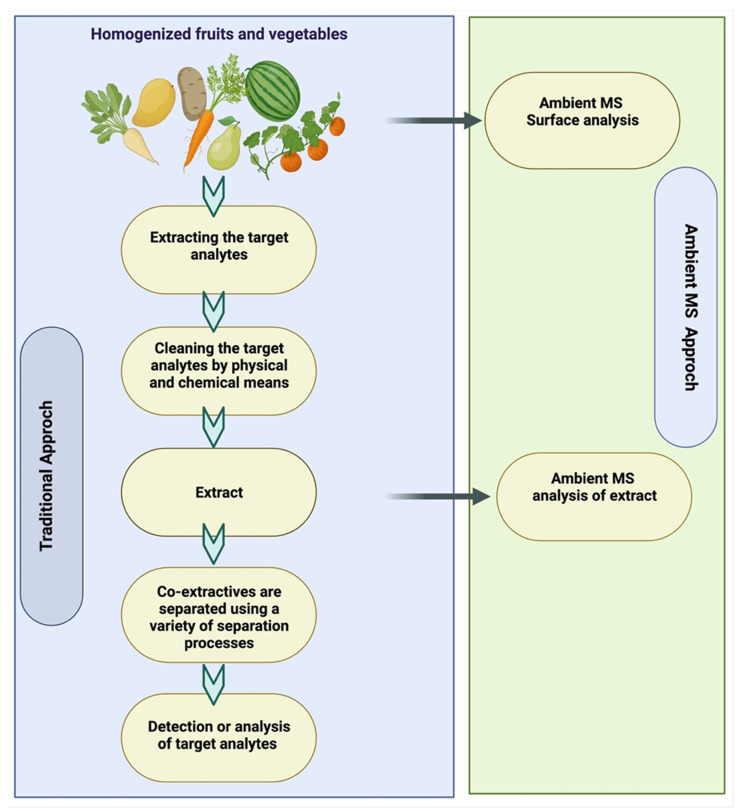
Ambient desorption/ionization mass spectrometry application.

Ambient mass spectrometry, which is used with condensed phase materials, is a set of processes that allows ions to be produced from samples under ambient conditions, which are then caught and evaluated using mass spectrometry. This method fits well with the need to quickly find pesticide residues in food for food safety testing. Ambient MS is a fast-emerging field that introduced desorption electrospray ionization (DESI) and direct analysis in real time [145,146]. Ambient desorption/ionization mass spectrometry application is shown in Figure 3.

Commercially accessible methods, such as direct analysis in real time (DART-MS), low-temperature plasma (LTP-MS), paper spray (PSMS), and desorption electrospray ionization (DESI-MS), are among the most often employed techniques. Fruit peel testing using DESI-MS has been proven to be a helpful screening approach for investigating materials carrying pesticides, either directly on the fruit peel surface or by scraping the peel using a glass slide and then using it as a DESI-MS substrate [147]. Chlorpropham was found on potato surfaces; dimethoate, tebuconazole, and trifoxystrobin were found on olive and vine leaves; and atrazine residues were found on Chinese cabbage leaves using DESI-MS. The existence of matrix effects and limited accuracy are key limits for quantitative analysis on surfaces [147,148,149]. The utilization of portable mass spectrometers to conduct in situ analysis is one of the most appealing characteristics of ambient ionization sources.

## 7. Impacts of Pesticide Residue Removal

Food safety is a rising challenge worldwide due to expanding population and the need to produce food to feed the growing population [150,151]. While pesticide usage increases agricultural output, pesticide residues over MRLs negatively impact animal and human health [132,152,153]. Pesticide residues found in high concentrations in fresh agriculture pose a significant threat to food safety. Further, using pesticides on horticultural crops pollutes the environment and leaves terminal degradation products in food, inducing teratogenic, carcinogenic, and immunosuppressive effects in human beings [154,155,156,157,158]. Evidence of more significant pesticide residues could be discovered [159]. In a study, pesticide residue of vegetables in Pakistan’s Hyderabad area was investigated. Results indicated that pesticides, including carbofuran and chlorpyrifos, widely used in agriculture were highly contaminated [160]. In vegetables and fruits, pesticides have high organochlorine and organophosphate levels [160,161,162,163]. These residues are increasingly ingrained in the food supply chain [25,164]. Carbendazim and neonicotinoids are widely used in fruits and vegetables to control sucking insects and fungi, and significant residues of these pesticides have been found in many countries [165,166,167,168,169]. These contaminants enter our food supply chain and negatively impact the human circulatory system after eating fresh vegetables and fruits. Numerous methods for reducing pesticide residues in vegetables and fruits have been investigated [170,171].

## 8. Possible Measures for Protecting People from These Contaminants in Food

Contaminated food is a cause of concern because of its harm to consumers’ health. These pesticides have been detected in raw and processed fresh fruits and vegetables. However, several studies have found that food processing techniques significantly reduce the pesticide residues in fruits and vegetables [172,173,174,175,176,177]. Like other meals, fruits and vegetables are processed before consumption. Pesticide residue levels in fruits and vegetables may be affected by where the pesticides are located and how they behave physically and chemically, such as how quickly they evaporate or dissolve, how much water they can be dissolved in, how quickly they break down, and how long they take to break down in the sun or heat [178]. According to public and scientific opinions, pesticide residues in food offer a greater danger to human health than other dietary concerns. Washing and boiling are some of the ways to get rid of these residues. In addition, boiling reduces pesticide residues in cabbage and cauliflower. Vegetables and fruits and their features impact the decrease in pesticide residues after washing [179]. The various food processing techniques that deal with the effect of pesticide residues have been summarized in Table 3. Pesticide residues are also affected by how the food is stored, handled, and processed between harvesting raw agricultural goods and eating ready-to-eat foods. The procedures utilized in the trials were mostly geared at commercial or home processing of fruits and vegetables, and they included boiling, frying, roasting, cooking, pureeing, peeling, blanching, and washing, among other methods of preparation. In order to acquire data that are meaningful, comparable, and transferable to different settings, processing and storage studies on pesticide residues in food are recommended. Pesticide residue levels were reduced in most food items due to processing processes, except when the substance was concentrated, such as juicing fruits and pressing or extracting oil from vegetable seeds.

## 9. Conclusions

Pesticides can cause adverse health effects on consumers, even at trace concentrations. Therefore, it is crucial to monitor pesticide residues in food items. Directly identifying all pesticides may be difficult due to the limited sensitivity of different detection technologies and matrix interference effects. Because of the complexities involved in determining pesticides, there is a need to improve existing techniques and develop new strategies to improve the reliability of analytical results. Regulations and limits can vary from country to country. The list of target compounds and matrices is constantly updated, making it difficult for food laboratories to choose methods and equipment to determine pesticide residues to keep up with the latest limits. The best sample preparation technique strikes a balance of cost, precision, selectivity, and sensitivity. Developing sample preparation procedures for detecting pesticide multi-residues in food samples is vital, since numerous physically and chemically diverse substances must often be assessed instead of just one or a specific class of analytes. Despite advances in detection technology, effective sample preparation procedures for pesticide residue measurement in cereals and feedstuffs are still needed. In addition, these methods must be compatible with current analytical techniques. For example, despite advancements in chromatographic separation and detection, cleaning is still required to acquire reliable results. According to the International Food Standards Organization, efficacious, ecologically friendly, and time-efficient technologies for sample treatment and pesticide residue assessment in fatty food matrices, such as cereals and feedstock, continue to be needed.

In the future, reliable, accessible, affordable, and eco-friendly sample preparation technologies should be adopted. High resolution, selectivity, and speed are key to expanding our analytical arsenal for these purposes to remain one step ahead of ever-lower regulated levels. New methods and instrumentation are thus essential. Furthermore, a short analytical turnaround time is critical to providing the required information for rapid and efficient pesticide residue testing in food matrices. To analyze more samples and ensure pesticide residues are within regulatory threshold limits, labs need increasingly sensitive triple quad mass spectrometers that deliver a lower cost per analysis, higher throughput, and decreased sample prep time.

Additionally, they should be comparable to commonly used analytical techniques to minimize expensive errors. Because of these reasons, analytical chemistry will make sample preparation more manageable and smaller and cut down on the size and amount of organic solvent used. In the future, new chromatographic–mass spectrometric techniques will be used to make it possible to test for pesticides at the low levels that are now required by law for many of them. This will make it possible to obtain more reliable data for food safety monitoring programs. This is the tendency, even though no one methodology can cover so many chemicals. Finally, multi-class multi-residue approaches that cover a wide range of pesticides are desired, even though pesticides’ diverse natures, classes, and physio-chemical characteristics make such methods challenging to assemble.

## Figures and Tables

**Table 1:** The maximum number of pesticides found in various fruits and vegetables.

Food Commodities	Number of Pesticide Residues
Strawberry	45
Apples	47
Grapes	56
Cherries	42
Tomatoes	35
potatoes	35
Sweet bell peppers	53

**Table 2:** The MRLs for fruits and vegetables in the United States, Canada, and Europe.

Pesticide Type	Example of Pesticide	European Commission ^1^				US-FDA ^2^				PCPA Canada ^3^			
		MRLs (µg kg^−1^)											
		Apple	Potato	Tomato	Strawberry	Apple	Potato	Tomato	Strawberry	Apple	Potato	Tomato	Strawberry
Carbamates	Propoxur	50	50	50	100	---	---	---	---	Banned			
	Aminocarb	---	---	---	---	---	---	---	---	---	---	---	---
	Carbofuran	1	1	2	50	---	---	---	---	---	500		400
	Carbaryl	10	10	10	50	12,000	2000	5000	4000	5000	200	5000	7000
	Propiconazole	150	10	300	50	---	---	3000	1300	---	---	3000	1300
Organo-phosphates	Parathion	50	50	50	100	---	---	---	---	Banned			
	Methyl parathion	10	10	10	50	---	---	---	---	Banned			
	Malathion	20	20	20	20	8000	8000	8000	8000	2000	500	3000	8000
	Diazinon	10	10	10	50	500	100	750	500	750		750	750
	Glyphosate	100	500	100	2000	200	200	100	200	---	---	---	---
Pyrethrins and pyrethroids	Deltamethrin	200	300	70	---	200	40	200	---	400	40	300	200
	Cypermethrin	1000	50	500	100	---	---	---	---	1000	100	300	200
	Permethrin	50	50	50	100	50	50	2000		1000	50	500	---
Organo-chlorines	Lindane	10	10	10	10	---	500	---	500	Banned			
	Captan	10^4^	30	100	100	25 × 10^3^	50	50	2 × 10^4^	5000	---	5000	5000
	Aldrin	10	10	10	10	30	100	50	50	---	---	---	---
	Chlordane	10	10	10	20	100	100	100	100	---	---	---	---
	Endosulfan	50	50	50	100	---	---	---	---	2000	---	1000	1000
	DDT	50	50	50	500	100	---	50	100	For fresh vegetables: 500			
	Dieldrin	10	10	10	10	30	100	50	50	---	---	---	---

Note: ^1^ EU Pesticides database. Retrieved from http://ec.europa.eu/food/plant/pesticides/eu-pesticides-database/public/?event=homepage&language=EN (accessed on 24 February 2022). ^2^ United States Department of Agriculture. Retrieved from https://www.fas.usda.gov/maximum-residue-limits-mrl-database (accessed on 24 February 2022). ^3^ MRLs for pesticides regulated under the Pest Control Products Act (PCPA). Retrieved from https://www.canada.ca/en/health-canada/services/consumer-product-safety/pesticides-pest-management/public/protecting-your-health-environment/pesticides-food/maximum-residue-limits-pesticides.html (accessed on 24 February 2022) [35].

**Table 3:** Summary of various food processing techniques dealing with the effect of pesticide residues.

Vegetables and Fruits	Pesticide Compounds	Operations	Conditions	Outcomes	References
Strawberries	Pyrimethanil Azoxystrobin Fenhexamid	Washing	The effect of ‘home’ washing with tap water and a commercially available vegetable detergent on residue levels was also studied.	Washing the fruit with tap water reduced the residues of azoxystrobin and fenhexamid but did not affect pyrimethanil residues. More significant amounts were removed when fruits were cleaned with a commercial detergent.	[180]
Peaches	Vinclozolin Procymidone Fenitrothion Chlorpyrifos-methyl	Washing Peeling Canning	Residues were determined in raw material.	Peeling was identified as the most effective procedure for reducing residues. However, thermal treatment (concentration and sterilization) substantially reduced residues.	[130]
Apricot	Diazinon, iprodione, procymidone, phosalone, and bitertanol	Sunlight- and oven-drying processes	Using sunlight and an oven to dry fruit made it more concentrated by about six times.	The sunlight treatment had more significant residue reductions than the oven procedure.	[181]
Tomatoes	Hexachlorobenzene (HCB), p,p-DDT, Lindane, Dimethoate, Profenos, Pirimiphos-methyl	Washing, Peeling, Juicing and Canning	Washing with acetic acid, sodium chloride, and tap water, freezing at −10 °C, juicing, peeling, and home canning at 100 °C for 30 min.	Washing with water or a detergent solution was necessary to decrease the intake of pesticide residues. In addition, freezing and juicing and peeling were essential to remove pesticide residues in the skin.	[182]
Tomatoes	Tralomethrin Pyridaben Pyrifenox	Washing Peeling Boiling	Residue levels in unprocessed and processed tomato samples were determined.	The washing processing factor results were 0.9 ± 0.3 for pyridaben, 1.1 ± 0.3 for pyrifenox, and 1.2 ± 0.5 for tralomethrin, whereas the peeling processing factors were 0.3 ± 0.2 for pyridaben and 0.0 ± 0.0 for both pyrifenox and tralomethrin.	[183]
Carrots, tomatoes	Captan Iprodione Mancozeb Metalaxyl Diazinon Endosulfan Parathion Cypermethrin Carbofuran	Washing Juicing	The distribution of nine pesticides between the juice and pulp of carrots and tomatoes during home culinary practices was investigated.	Washing of the produce removed more residue from carrots than from tomatoes, but it did not affect the relative distribution of the residues.	[184]
Peaches, oranges, Broccoli, cabbage, green beans, Winter squash, sweet potatoes, apples, cherries, peppers	3,5,6-Trichloro-2-pyridinol Chlorpyrifos	Juicing Canning Boiling Baking	The fate of the residues of benalaxyl, dimethoate, iprodione, metalaxyl, phosalone, procymidone, and vinclozolin in sunlight and oven raisin processing was studied.	Sunlight-drying was more effective for phosalone and vinclozolin, whereas oven-drying was more effective for iprodione and procymidone due to the washing effect rather than dehydration.	[185]
Apricot	Dimethoate, fenitrothion, ziram, omethoate	Sunlight and ventilated oven drying	Samples warm for 30 min at 100 °C and 12 h at 70 °C.	The half-lives of the pesticides ranged from 6.9 to 9.9 days, with pseudo-first-order kinetics and degradation rates of 6.9 to 9.9 days.	[186]
Spearmint, caraway, anise Lindane Chamomile, karkade	Lindane, Profenos, DDT, Pirimiphos-methyl, Endrin,	Boiling	2 g of the dry plant were left to boil in 100 mL deionized water for 5 min in a glass beaker. In the second method, 2 g of the dry sample was immersed in 100 mL of hot deionized water for 5 min (tea method).	Residues were not detected in the watery extract when the medicinal plant was boiled in water. Moreover, immersing the plants in hot water transferred pesticide residues to the aqueous extract.	[187]
Apple	Phosalone	Rotating ‘Hatmaker’ drum dryer	Steam pressure (5 bars), discharge rate (150 L/h), rotation speed (5–76 cm/s)	Phosalone levels were reduced from 22 to 77%. Manufacturers should seek the total elimination of surface residues, i.e., peeling the fruit to improve quality.	[188]
Apple pomace	kelthane	Apple pomace exposed to drying in the dark, sunlight and ultraviolet light irradiation	In the dark, under UV light or sunlight	The loss of kelthane residues was mainly due to volatility rather than photodecomposition.	[189]
Honeysuckle (Lonicera japonica)	Thiacloprid and thiamethoxam	Planting, drying, and tea brewing processes	Oven-drying at 30, 40, 50, 60, and 70 °C	Drying methods and tea brewing conditions can reduce the transfer of thiamethoxam and thiacloprid to humans.	[190]
Chili pepper	Tetraconazole, methoxyfenozide, clothianidin, diethofencarb, methomyl, indoxacarb, imidacloprid, diethofencarb, and chlorfenapyr	Oven drying	60 °C for 35 h	Clothianidin, diethofencarb, imidacloprid, and tetraconazole reductions (37–49%). Moderate decreases in methomyl (16%) and methoxyfenozide (22%). Indoxacarb and folpet levels were unaffected by drying.	[191]
Jujube	Cyhalothrin, bifenthrin, epoxicona-zole, tebuconazole, kresoxim-methyl, myclobutanil, hexaconazole, triadimefon, chlorpyrifos, malathion, dichlorvos	Drying by microwave	Microwave oven (700 W) for 4 min	The degradation rates ranged from 67% to 93%.	[192]
Okra	Profenofos, bifenthrin	sun drying	No specific conditions were found	Profenos up to 11% and bifenthrin, up to 75%. Bifenthrin was more affected by sun-drying because it is hydrolyzed in the presence of UV rays.	[193]
Okra	Carbaryl, malathion, endosulfan	Convective drying	No specific conditions were found	78% carbaryl, 91.8% malathion, and 57.4% endosulfan removal and sun-drying helped decrease endosulfan up to 5.5%.	[194]
*Pleurotus ostreatus* mushroom	Carbendazim	freeze-drying and sun drying	Direct sunlight (sun drying) and at −86 °C with a vacuum of 0.06 mbar (freeze-drying).	Direct sun-drying removed higher carbendazim amounts than freeze-drying, with removal rates ranging between 70 and 97%.	[195]
Kumquat candied fruit	Triazophos, chlorpyrifos, malathion, methidathion, and dimethoate	Convective drying	60–80 °C	Dimethoate, malathion, and triazophos had PF values more significant than one upon drying, which might be attributed to water loss.	[196]
Grape	Dimethoate, diazinon, chlorpyrifos, and methidathion	Oven and sun drying	Direct sunlight for 21 days and in an oven at 50 °C for 72 h, at 60 °C for 60 h, at 70 °C for 48 h, at 80 °C for 36 h	The greater the temperature, the faster pesticides degrade in grape drying processes.	[197]
Plum	Vinclozolin, procymidone, iprodione, diazinon, and bitertanol	Oven drying	Temperature: 30 min at 95 °C, 30 min at 90 °C, 16 h at 85 °C	Procymidone, iprodione, and bitertanol were lower in dried fruits than fresh fruits (0.6, 2.3, and 3.2 times, respectively).	[198]
Spring onion	Etofenprox	Drying	Freeze-dried (3 days) and the oven (80 °C for 24 h).	Oven-dried has a greater removal rate (85.5 percent) than freeze-dried (66.6 percent).	[199]
Shiitake mushroom	β-cyfluthri, λ-cyhalothrin, bifenthrin, procymidone, thiabendazole, carbendazim	Drying	Sunlight (26–33 °C, 20 days) and hot-air drying (30–53 °C in the first 10 h, 53–60 °C in the last 10 h)	The removal rate of pesticides by sunlight exposure drying (36.2–94.6%) was higher than that of hot-air drying (26.0–68.1%).	[200]
Red pepper	Fenitrothion and chlorpyriphos	Hot air drying and sun drying	No specific conditions were found	20–30 percent of residues were removed by drying in the sun or hot air.	[193,201]

## Data Availability

Data sharing is not applicable to this article.
